# Tonotopic and Field-Specific Representation of Long-Lasting Sustained Activity in Rat Auditory Cortex

**DOI:** 10.3389/fncir.2016.00059

**Published:** 2016-08-10

**Authors:** Tomoyo I. Shiramatsu, Takahiro Noda, Kan Akutsu, Hirokazu Takahashi

**Affiliations:** ^1^Research Center for Advanced Science and Technology, The University of TokyoTokyo, Japan; ^2^Technical University of MunichMunich, Germany; ^3^Graduate School of Information Science and Technology, The University of TokyoTokyo, Japan

**Keywords:** auditory cortex, auditory evoked potential, sustained activity, phase locking value, microelectrode array, machine learning, sparse logistic regression

## Abstract

Cortical information processing of the onset, offset, and continuous plateau of an acoustic stimulus should play an important role in acoustic object perception. To date, transient activities responding to the onset and offset of a sound have been well investigated and cortical subfields and topographic representation in these subfields, such as place code of sound frequency, have been well characterized. However, whether these cortical subfields with tonotopic representation are inherited in the sustained activities that follow transient activities and persist during the presentation of a long-lasting stimulus remains unknown, because sustained activities do not exhibit distinct, reproducible, and time-locked responses in their amplitude to be characterized by grand averaging. To address this gap in understanding, we attempted to decode sound information from densely mapped sustained activities in the rat auditory cortex using a sparse parameter estimation method called sparse logistic regression (SLR), and investigated whether and how these activities represent sound information. A microelectrode array with a grid of 10 × 10 recording sites within an area of 4.0 mm × 4.0 mm was implanted in the fourth layer of the auditory cortex in rats under isoflurane anesthesia. Sustained activities in response to long-lasting constant pure tones were recorded. SLR then was applied to discriminate the sound-induced band-specific power or phase-locking value from those of spontaneous activities. The highest decoding performance was achieved in the high-gamma band, indicating that cortical inhibitory interneurons may contribute to the sparse tonotopic representation in sustained activities by mediating synchronous activities. The estimated parameter in the SLR decoding revealed that the informative recording site had a characteristic frequency close to the test frequency. In addition, decoding of the four test frequencies demonstrated that the decoding performance of the SLR deteriorated when the test frequencies were close, supporting the hypothesis that the sustained activities were organized in a tonotopic manner. Finally, unlike transient activities, sustained activities were more informative in the belt than in the core region, indicating that higher-order auditory areas predominate over lower-order areas during sustained activities. Taken together, our results indicate that the auditory cortex processes sound information tonotopically and in a hierarchical manner.

## Introduction

During the perception of sensory objects, the sensory cortex integrates two types of information about the target object: the boundary from the environment and the continuous body surrounded by that boundary. For instance, a simple acoustic stimulus consists of an onset, continuous plateau, and offset. Accumulating evidence indicates that during object perception, the auditory cortex processes information from all components of the stimulus (Heil, 1997; Takahashi et al., 2004; Petkov et al., 2007; Riecke et al., 2009; Vinnik et al., 2012). Under normal circumstances, neural systems are sensitive to rapid changes in sensory input; these systems increase the signal-to-noise ratio of transient activities in response to the onset and offset of sound stimuli and adapt after hundreds of milliseconds. Therefore cortical representation (e.g., cortical subfields and topographic representation in these subfields, such as place code of sound frequency) have been well characterized from onset activities (Doron et al., 2002; Rutkowski et al., 2003; Takahashi et al., 2004; Polley et al., 2007; Noda and Takahashi, 2015). However, sustained activities that follow transient activities and persist during the presentation of a continuous stimulus have received less attention because these activities do not exhibit distinct, reproducible, and time-locked responses in their amplitude to be extracted clear neural features by grand averaging across trials. Therefore, although sustained activities should play an important role in acoustic object perception, their neural representations remain unclear.

Previously, it has been reported that the neural synchrony of unreproducible sustained activities lasting hundreds of milliseconds to several seconds, usually called steady-state activities, represents sound information topographically in the auditory cortex (DeCharms and Merzenich, 1996; Eggermont, 1997). Yet, until recently, few studies have compared the cortical representation of steady-state and long-lasting sustained activities with that of transient activities, possibly because the optimal characteristics of these activities have not been identified and because it is technically demanding to densely map the neural synchrony of sustained activities in the auditory cortex. Recently, a microelectrode array with a 10 × 10 grid of recording sites enabled dense recording from the rat auditory cortex (Noda and Takahashi, 2015), allowing us to characterize neural synchrony (e.g., phase synchrony or PLV) of sustained activities between any two recording sites. However, an extremely high dimension of such characteristics makes it difficult to identify informative pairs of recording sites that represent sound information. In our recent studies, we attempted to address this problem by using machine learning (e.g., SLR). As a machine learning algorithm for classification, SLR can be applied to high-dimensional data, even when the dimension exceeds the number of samples (Yamashita et al., 2008). We demonstrated that SLR could classify the densely mapped band-specific power and PLV of sustained activities for each test frequency (Shiramatsu et al., 2013, 2014). Here, as a next step, we focused on the ‘feature selection’ or ‘sparse parameter estimation’ of SLR and attempted to reveal the cortical representation of these characteristics in sustained activities. During supervised learning for classification, SLR prunes irrelevant dimensions of data by setting their associated weights to zero and is most effective when input data has sparse representation, like those in neural activity patterns. Recent studies demonstrated that selected features in SLR or other regression framework indicated some possible neural correlates (Chao et al., 2010; Yahata et al., 2016). Thus, we assumed that the selected recording sites, or informative recording sites, will contribute to revealing the cortical representation of sustained activities.

In this study, we investigated whether and how sustained activities represent sound information by decoding sound information from densely mapped neural activities in the rat auditory cortex with sparse parameter estimation (e.g., SLR). A microelectrode array with a 10 × 10 grid of recording sites was implanted to record transient and sustained activities from the fourth layer of the auditory cortex of anesthetized rats. SLR was applied to discriminate sound-induced band-specific power and PLV from those of spontaneous activities, and we investigated the CF and cortical subfield of the informative recording sites. In addition, to examine the tonotopic representation of sustained activities, we tested whether decoding accuracy decreased when SLR was used to discriminate close test frequencies.

## Materials and Methods

This study was carried out in strict accordance with the “Guiding Principles for the Care and Use of Animals in the Field of Physiological Science” published by the Japanese Physiological Society. The experimental protocol was approved by the Committee on the Ethics of Animal Experiments at the Research Center for Advanced Science and Technology at the University of Tokyo (Permit Number: RAC130107). All surgeries were performed under isoflurane anesthesia, and every effort was made to minimize suffering. After the experiments, animals were euthanized with an overdose of pentobarbital sodium (160 mg/kg, i.p.).

### Electrophysiological Experiments

#### Animal Preparation

Ten Wistar rats (postnatal weeks 9–11, body weight of 270–310 g) were used in the study. Rats were anesthetized with isoflurane in conjunction with air (3% for induction and 1–2% for maintenance), and held in place with a custom-made head-holding device. Atropine sulfate (0.1 mg/kg) was administered at the beginning and end of the surgery to reduce the viscosity of bronchial secretions. A heating blanket was used to maintain body temperature at approximately 37°C. A skin incision was made at the beginning of the surgery under local anesthesia (1% xylocaine, 0.3–0.5 ml). A needle electrode was subcutaneously inserted into the right forepaw, and used as a ground. A small craniotomy was performed near bregma to embed a 0.5-mm thick integrated circuit socket as a reference electrode with an electrical contact to the dura mater. The right temporal muscle, cranium, and dura overlying the auditory cortex were surgically removed and the exposed cortical surface was perfused with saline to prevent desiccation. Cisternal cerebrospinal fluid drainage was performed to minimize cerebral edema. The right eardrum (i.e., ipsilateral to the exposed cortex) was ruptured and filled with wax to ensure unilateral sound inputs from the ear contralateral to the exposed cortex. Respiratory rate, heart rate, and hind-paw withdrawal reflexes were monitored throughout the experiment to maintain an adequate and stable anesthetic level.

#### Neural Recordings

A microelectrode array (ICS-96, Blackrock Microsystems, Salt Lake City, UT, USA) with a 10 × 10 grid of recording sites within an area of 4 mm × 4 mm simultaneously recorded LFPs from the fourth layer of the auditory cortex, i.e., 600 μm in depth (Noda et al., 2013). LFPs were obtained with an amplification gain of 1,000, digital filter bandpass of 0.3–500 Hz, and sampling frequency of 1 kHz (Cerebus Data Acquisition System, Cyberkinetics Inc., Salt Lake City, UT, USA). MUAs were obtained with an amplification gain of 1,000, digital filter bandpass of 250–7,500 Hz, and sampling frequency of 30 kHz. From this signal, multi-unit spikes were detected by threshold-crossing (set to -5.65 times the root mean square of the signal) during online processing. Four recording sites at the corners of the grid were offline, thus 96 recording sites were used for recording. A function generator (WF1947; NF Corp., Kanagawa, Japan) was used to generate acoustic stimuli. A speaker (Technics EAS-10TH800, Matsushita Electric Industrial Co. Ltd., Osaka, Japan) was positioned 10 cm from the left ear (i.e., contralateral to the exposed cortex). Test stimuli were calibrated at the pinna with a 1/4-inch microphone (4939, Brüel & Kjær, Nærum, Denmark) and spectrum analyzer (CF-5210, Ono Sokki Co., Ltd., Kanagawa, Japan). The stimulus level is presented in dB SPL (sound pressure level in decibels with respect to 20 μPa).

First, we recorded transient activities responding to pure tone bursts. Test stimuli were pure tone bursts (5 ms rise/fall and 20 ms plateau) with frequencies from 1.6 to 64 kHz (1.6, 2.0, 2.5, 3.2, 4.0, 5.0, 6.4, 8.0, 10, 13, 16, 20, 25, 32, 40, 50, 57, and 64 kHz) and intensities from 20 to 80 dB SPL in 10-dB increments; 126 different tone bursts were tested in total. Each test tone was repeated 20 times in a pseudorandom order with an inter-tone interval of 600 ms. We recorded LFPs as transient activities, and MUA to characterize the FRA and spike peak latency at each recording site.

**FIGURE 1:**
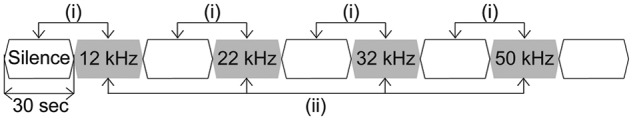
**Schematic diagram of long-lasting pure tone stimuli used in this study.** Long-lasting pure tones with a duration of 30 s, including a 5-ms rise/fall, with an intensity of 60 dB SPL and frequencies from 12 to 50 kHz **(Table 1)**. Each of the pure tones was presented in a pseudorandom order and interleaved with a silent block of 30 s.

Next, we recorded LFPs as sustained activities responding to long-lasting pure tones (30-s duration including 5 ms rise/fall, 60 dB SPL) with frequencies from 12 to 50 kHz (**Table 1**). Each of the pure tones was repeated 7–12 times in a pseudorandom order and interleaved with a 30-s silent block (**Figure 1**).

**Table 1:** Test frequencies used in the experiments.

	Frequencies #1, kHz	Frequencies #2, kHz	Frequencies #3, kHz	Frequencies #4, kHz	Frequency range, octave
Set #1	12	22	32	50	2.1
Set #2	12	15	19.2	24	1
Set #3	14.4	16	18	20	0.47
Set #4	13.5	14.4	15	16	0.25


*For each set of frequencies, SLR decoded four test frequencies from sustained activities. The frequency range is indicated in the rightmost column.*

#### Characterization of the Recording Sites

Data analysis was carried out in MATLAB (Mathworks, Natick, MA, USA). From the MUA in response to pure tone bursts, we characterized the FRA and spike peak latency at each recording site. To characterize the FRA, the number of tone-evoked spikes was defined for each test tone by subtracting the mean firing rate within 1–600-ms post-stimulus latency from that within 5–55 ms. The number of spikes in response to each tone was then normalized using the maximum number of MUAs among all test tones. The FRA then was characterized at each recording site as a map of normalized spike rates in the frequency-intensity plane of test tones. A CF was determined to be the frequency at which test tones evoked the largest response at the threshold or at 20 dB SPL (i.e., the minimum intensity used in this experiment). In addition, at each recording site, PSTH were obtained in 1-ms bins from the MUA in response to 80-dB CF tones, and spike peak latency was defined as the time when the PSTH exhibited a maximum.

### Machine Learning

Three characteristics were extracted from the recorded LFPs: amplitude of the evoked potential from transient activities and band-specific power and PLV from the sustained activities. SLR was applied to decode test frequencies from these characteristics. First, SLR discriminated sound-induced activities from spontaneous activities to demonstrate (1) whether informative recording sites are consistent with the tonotopic structure as determined from the MUAs, and (2) whether the core or belt region is more informative in the decoding. Second, we tested whether decoding accuracy decreases when SLR discriminates several test frequencies within a narrower test frequency range (**Table 1**).

#### Calculation of the Characteristics of Neural Activity

##### Characteristics of the transient activities

**FIGURE 2:**
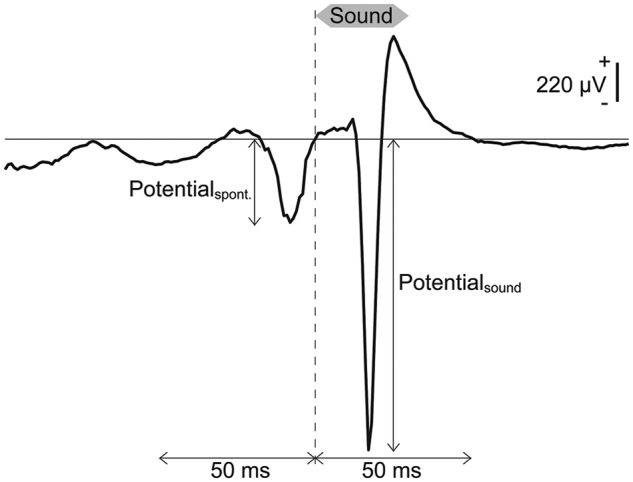
**Transient activities in response to pure tones based on LEPs.** Representative LFP obtained from a pure tone burst with a frequency of 50 kHz and an intensity of 70 dB SPL. Spontaneous potentials and AEPs were quantified as the absolute values of the minimum peak in the 50-ms LFPs preceding and following the onset of each pure tone burst.

It is well known that AEPs, in response to sound onset exhibit tonotopically organized spatial distributions from which machine learning can discriminate test frequencies (Shiramatsu et al., 2013, 2014). Thus, SLR first discriminated AEPs from the spontaneous potentials. At each recording site, the amplitude of the middle-latency AEP, termed P1, was quantified as the absolute value of the minimum peak of the single-trial LFP within 50 ms from the onset of the pure tone bursts (13, 20, 32, and 50 kHz at 50, 60, and 70 dB SPL) at each recording site (**Figure 2**). Spontaneous potentials also were quantified as the absolute values of the minimum peak in the 50-ms LFPs preceding each pure tone burst (**Figure 2**). Each frequency-intensity pair was presented 20 times for a total of 60 samples for the AEPs and 60 samples for the spontaneous potentials for each sound frequency, and the dimension of each sample was 96 (i.e., the number of the recording sites).

##### Characteristics of the sustained activities

**FIGURE 3:**
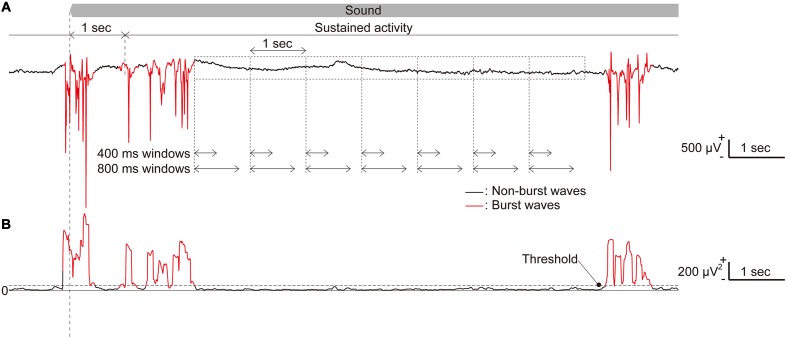
**Long-lasting sustained activities.** **(A)** Representative raw traces of long-lasting, sustained LFPs in response to a pure tone with a frequency of 50 kHz. **(B)** The standard deviation (SD) of a 100-ms LFP, where burst activities were included. Time periods, during which the SD exceeds a threshold in more than 24 recording sites and with durations of 150 ms or longer, were classified as burst LFPs (red line) while others were classified as non-burst LFPs (black line). The time window length used in the analyses was chosen from five tested durations.

It has been reported that isoflurane anesthesia often produces a pattern of alternating high amplitude bursts and suppressed activity regardless of the sound presentation (Land et al., 2012; **Figure 3A**). This burst activity simultaneously appears in almost all recording sites of the microelectrode array, and our previous study demonstrated that SLR could not decode the test frequency from the characteristics in such burst activity (Shiramatsu et al., 2014). Thus, we first eliminated bursting LFPs from the analysis using the following methods. First, the standard deviation (SD) of each 100-ms LFP was calculated at each time point (**Figure 3B**). The threshold to determine burst activities was calculated as the sum of the average and three times the SD of these SDs in part of the recorded data. This threshold was calculated for each rat. This threshold was then used to categorize all recorded LFPs into burst and non-burst waves (**Figure 3**). First, the SD of the LFP at each time point was classified as a burst wave if the SDs at more than 24 recording sites exceeded the threshold. Then, the burst LFPs with durations shorter than 150 ms were eliminated to reduce the false positive detection of burst waves.

After classifying burst and non-burst waves, we examined five different time-window lengths for analysis. In this study, we defined sustained activities as neural activities 1–29 s from the sound onset (sound-induced activity) or from the sound offset (spontaneous activity) to eliminate the effect of onset and offset activities. First, we extracted 100 or 60 time periods of 1,000 ms shifted by 1,000 ms from each sound-induced and spontaneous sustained activity. From these time periods, we obtained time windows with five different lengths [200, 400, 600, 800, and 1,000 ms (Shiramatsu et al., 2013)] from the beginning of each time period (**Figure 3B**).

From the LFPs within these time windows, we extracted band-specific power and PLV in five bands (theta, 4–8 Hz; alpha, 8–14 Hz; beta, 14–30 Hz; low-gamma, 30–40 Hz; and high-gamma, 60–80 Hz). Band-specific power was calculated as the root mean square of the bandpass-filtered LFPs within the time windows at each recording site. Within each time window, PLVs between all 4,560 pairs of 96 recording sites were calculated according to the following equation (Doesburg et al., 2008):

$${PLV}_{\left(j,k\right)}=\frac{1}{T}\times |\underset{t=T}{\Sigma }e^{i\left\{\theta_{j}(t)-\theta_{k}\left(t\right)\right\}}|$$

where *j* and *k* indicate the recording site number, theta indicates the instantaneous angle at each time obtained by the Hilbert transform of the filtered LFP, *T* indicates the time included in the time window, and *i* is the imaginary unit. PLV is a real value between 0 and 1. When band-passed LFPs at two recording sites were perfectly synchronized or their phase difference was constant during a time window, PLV was 1; and when they were completely desynchronized or their phase difference was completely random, PLV was 0.

#### Decoding of Test Frequencies

Sparse logistic regression (Yamashita et al., 2008; Ryali et al., 2010) was applied to decode the test frequency from the three characteristics of neural activities, as indicated above in section “Machine Learning.” For this decoding procedure, SLR toolbox ver1.2.1 alpha was used as a toolbox for MATLAB.

##### Discrimination of sound-induced activities from spontaneous activities

**FIGURE 4:**
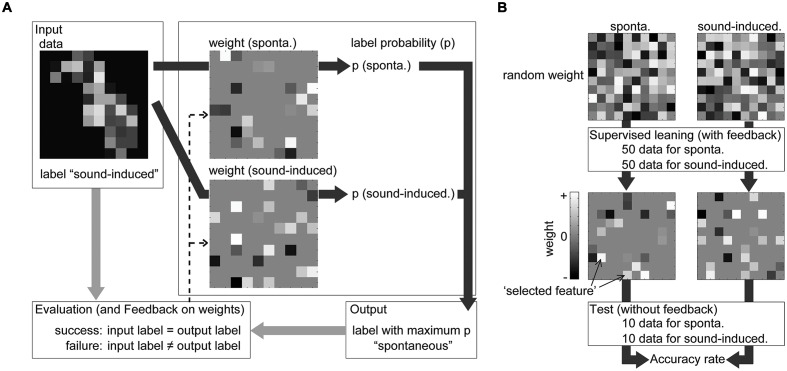
**Discrimination by SLR during supervised learning and the subsequent testing process.** **(A)** From one input, SLR calculated and compared label probability (indicated by deep gray arrows). SLR had weight vectors equal to the tested label (in this figure, two labels) and the input value at each recording site was multiplied with the weight at the same recording site. From the summation of this multiplication of the input data and weight vectors, the softmax function calculated the ‘label probability (p)’ for each label. SLR then determined the label with the maximum label probability as the output label or discrimination result. In supervised learning, SLR renewed the weight vector (indicated by the dotted arrows) according to the comparison between the input and output label (indicated by light gray arrows). **(B)** The discrimination consisted of two steps: supervised learning and the testing process. After supervised learning, SLR again discriminates new samples and we obtained the accuracy rate of the discrimination and weight vector for each label.

Sparse logistic regression was applied to discriminate sound-induced activities from spontaneous activities and extract informative recording sites (**Figure 4**). To discriminate transient activities from spontaneous activities, the feature vector of AEP (96 dimensions, 60 samples) or spontaneous potential (96 dimensions, 60 samples) was labeled as ‘sound-induced’ or ‘spontaneous’ activity, and divided into six subgroups. As shown in **Figure 4B**, the discrimination consisted of two steps: supervised learning with five labeled subgroups and the testing process with the remaining subgroup. In both steps, SLR had weight vectors as many as the tested label (in this case, two), SLR first summed the multiplication of the input data and weight vectors at the recording site for each label, and softmax function calculated ‘label probability’ for each label [**Figure 4A**, for details, see (Yamashita et al., 2008)]. SLR then determined the label with the maximum label probability as the output label or discrimination result. In supervised learning, SLR renewed the weight vector according to the comparison between input and output label (**Figure 4A**, dotted line). In this feedback process, SLR set the weight at non-informative recording site to zero, thus after supervised learning with 100 samples (50 for each label), most of the elements of the weight vector were zero (**Figure 4B**). In the testing process after supervised learning, SLR again discriminates 20 samples (10 for each label), and we obtained accuracy rate of the discrimination as the percentage of the successfully discriminated samples (**Figure 4B**). This process was repeated six times for each test frequency and rat. Then, we calculated the mean accuracy rate among the six cross-validations and four test frequencies in each rat, and compared it with the chance level, e.g., 50%, to evaluate the performance of the discrimination.

To discriminate sustained activities from preceding spontaneous activities [**Figure 1** (i)], the feature vector of band-specific power (96 dimension, 100 samples) or PLV (4560 dimension, 100 samples) was labeled as ‘sound-induced’ or ‘spontaneous’ activity, and divided into 10 subgroups. A 10-fold cross-validation was applied [e.g., supervised learning with 180 samples (90 samples for each label) and test process with 20 samples (10 for each label)] for each test frequency, frequency band of the filter, time window length, and rat. The mean accuracy rate among the ten cross-validations and four test frequencies was calculated in each frequency band, time window length and rat, and compared with the chance level.

##### Decoding of test frequencies in several frequency ranges

We tested whether sustained activities could be decoded within a narrow test frequency range, which was expected to impair the decoding performance. The feature vector of the 1,000-ms high-gamma power or PLV (60 samples for each label and condition) was labeled as one of the four test frequencies [**Table 1** and **Figure 1** (ii)]. A sixfold cross validation [e.g., supervised learning with 200 samples (50 samples for each label) and test process with 40 samples (10 for each label)] was used for each set of test frequencies, frequency band, time window length, and rat. The accuracy rate across the label and cross-validation in each animal and frequency range was compared.

## Results

### Discrimination of Sound-Induced Activities from Spontaneous Activities

#### Accuracy Rate of SLR for the Discrimination

**FIGURE 5:**
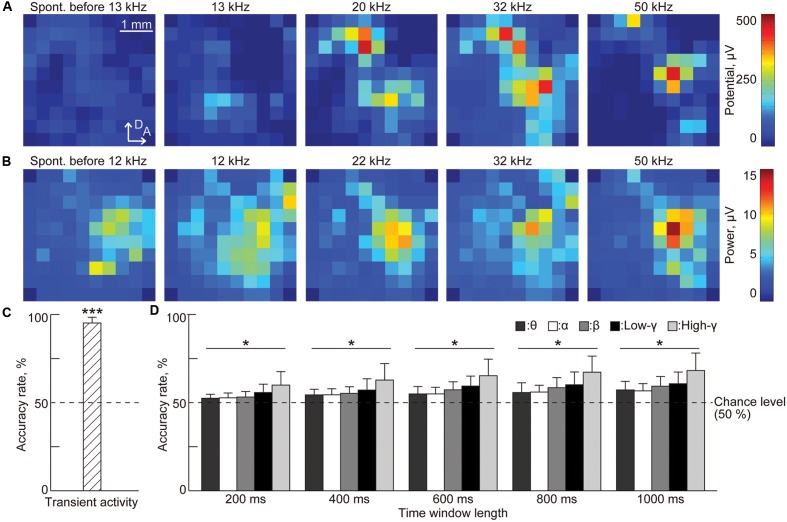
**Sparse logistic regression decoding from transient activities and the band-specific power of sustained activities.** **(A)** Representative spatial patterns of transient potentials in spontaneous activity (leftmost) and in response to varied test frequencies: 13, 20, 32, and 50 kHz. **(B)** Representative spatial patterns of high-gamma power for 1,000 ms in spontaneous activity (leftmost) and in response to varied test frequencies: 12, 22, 32, and 50 kHz. **(C,D)** Decoding accuracy **(C)** in transient activities and **(D)** in band-specific power. For band-specific power, both the bands and the time window length were varied. Asterisks indicate that decoding performance was better than the chance level, i.e., 50%: ^∗∗∗^*p* < 0.001 (Wilcoxon signed-rank test), ^∗^*p* < 0.05 (Wilcoxon signed-rank test). Spont., spontaneous activity.

To demonstrate whether the three characteristic, i.e., the amplitude of the evoked potential from transient activities, the band-specific power and the PLV from sustained activities (see Materials and Methods), represent sound information, we examined the accuracy rate in the testing process of SLR. **Figure 5A** shows the representative spatial patterns of the potentials in spontaneous activity and the AEPs in transient activities. Spontaneous activity did not exhibit large potentials. In contrast, AEPs exhibited several distinct activation foci, depending on the test frequency. Because there are clear differences between auditory-evoked and spontaneous potentials, the accuracy rate was high at 95.3%, which is significantly better than the chance level [**Figure 5C**, *n* = 8 (rats), *p* < 0.001 (Wilcoxon signed-rank test)].

Both sound-induced sustained activity and spontaneous activity exhibited high band-specific power at some recording sites that makes these activities more difficult to discriminate based on band-specific power (**Figure 5B**). Nonetheless, discrimination accuracy increased with the length of the time window and the highest accuracy (68.3%) was achieved in the high-gamma band for a time window of 1,000 ms [**Figure 5D**, *n* = 8 (rats), *p* < 0.05 (Wilcoxon signed-rank test)].

**FIGURE 6:**
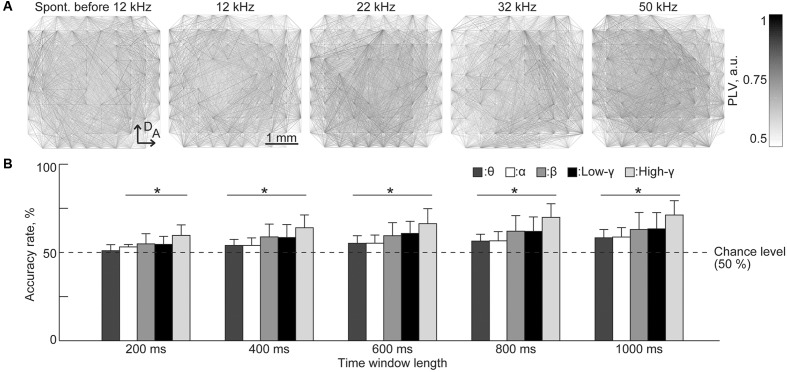
**Sparse logistic regression decoding from the PLVs of sustained activities.** **(A)** Representative spatial pattern of high-gamma PLV for 1,000 ms in spontaneous activity (leftmost) and in response to varied test frequencies: 12, 22, 32, and 50 kHz. **(B)** Decoding accuracy. Bands and the duration of the time window served as parameters. Asterisks indicate that the decoding performance was better than the chance level, i.e., 50%: ^∗^*p* < 0.05 (Wilcoxon signed-rank test). Spont., spontaneous activity.

Finally, PLV patterns have extremely high dimensionality, e.g., 4,560 dimensions in our case, and representative patterns indicate that it is difficult to extract distinct patterns or informative pairs of recording sites that represent the existence of a test tone (**Figure 6A**). Surprisingly, the SLR was able to discriminate between sound-induced and spontaneous activities from PLV patterns with the highest accuracy (71.3%) achieved in the high-gamma band for the time window of 1,000 ms [**Figure 6B**, *n* = 8 (rats), *p* < 0.05 (Wilcoxon signed-rank test)].

#### Characteristic Frequency and Subregions of the Informative Recording Sites

##### Characterization of the auditory cortex based on characteristic frequency and spike peak latency

**FIGURE 7:**
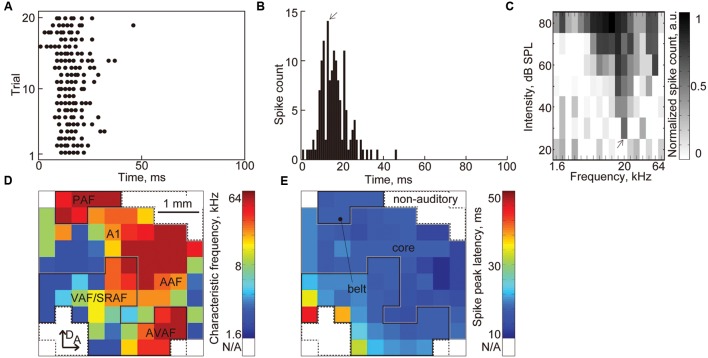
**Characterization of the auditory cortex based on the CF and spike peak latency.** **(A)** Representative raster plot. The black dots indicate MUAs. **(B)** PSTH of the raster plots in **(A)**. The arrow indicates the peak latency. **(C)** FRA at each recording sites. The arrow indicates the CF (20 kHz) at this recording site. **(D)** Tonotopic map and **(E)** latency map in the auditory cortex. Color map of the CF and spike peak latency at each recording site is shown. From these maps, we classified each recorded site into three functional regions: core, belt, and non-auditory region. The recording sites that had no clear CF were categorized in the non-auditory region. The borders between the core and belt regions were determined from the tonotopic gradient and spike peak latency.

We characterized the FRA and spike-peak latency at each recording site (**Figures 7A–C**), and from the maps of the FRA and spike-peak latency (**Figures 7D,E**), we classified each recorded site into one of three functional regions: core, belt, and non-auditory region (Doron et al., 2002; Rutkowski et al., 2003; Polley et al., 2007; Takahashi et al., 2011; Noda and Takahashi, 2015). First, the recording sites that had no clear CF were categorized in the non-auditory region. Then, the borders between the core and belt regions were determined from the tonotopic gradient and the spike-peak latency. The core region consists of the A1 and the AAF, each of which exhibits a posterior-to-anterior or lateral-to-dorsal tonotopic gradient and short peak latency (**Figure 7D**). The belt region consists of five subfields: the ventral/suprarhinal auditory fields (VAF/SRAF), PAF, DAF, and AVAF. The VAF/SRAF was characterized by three features: location ventral to A1 and posterior to AAF, tonotopic gradient along the posteroventral-to-anterodorsal axis, and longer latencies than the core region (**Figure 7E**). The PAF, DAF, and AVAF were determined by two features: the relative location to the core region (PAF, dorsal to A1; DAF, dorsal to the CF-reversal point in the core region; AVAF, ventral to AAF) and the discontinuation of the CF from the core region (PAF, relatively high CF; DAF, relatively low CF; AVAF, high CF). The recording sites in these putative two or five subfields were categorized in the core and belt regions, respectively.

##### Characteristic frequency and subregions of the informative recording sites

During the supervised learning phase, SLR selected informative recording sites and removed irrelevant recording sites from the discriminator by updating their weights (**Figure 4B**; for representative activity at the informative and non-informative sites, please see **Supplementary Figures S1** and **S2**). We were interested in determining (1) whether the informative recording sites reflect tonotopic organization in the auditory cortex, and (2) whether the core or belt region is more informative in the discrimination. To address these questions, we examined the remaining features, i.e., recording sites with non-zero weights after decoding, in the most successful 20 discriminations of each test frequency. For sustained activities, we only investigated the remaining features in the discrimination from high-gamma band-power and PLV in the 1,000-ms time window length, where the highest decoding performance was achieved. The results in other frequency bands were shown in **Supplementary Figures S3**–**S5**.

**FIGURE 8:**
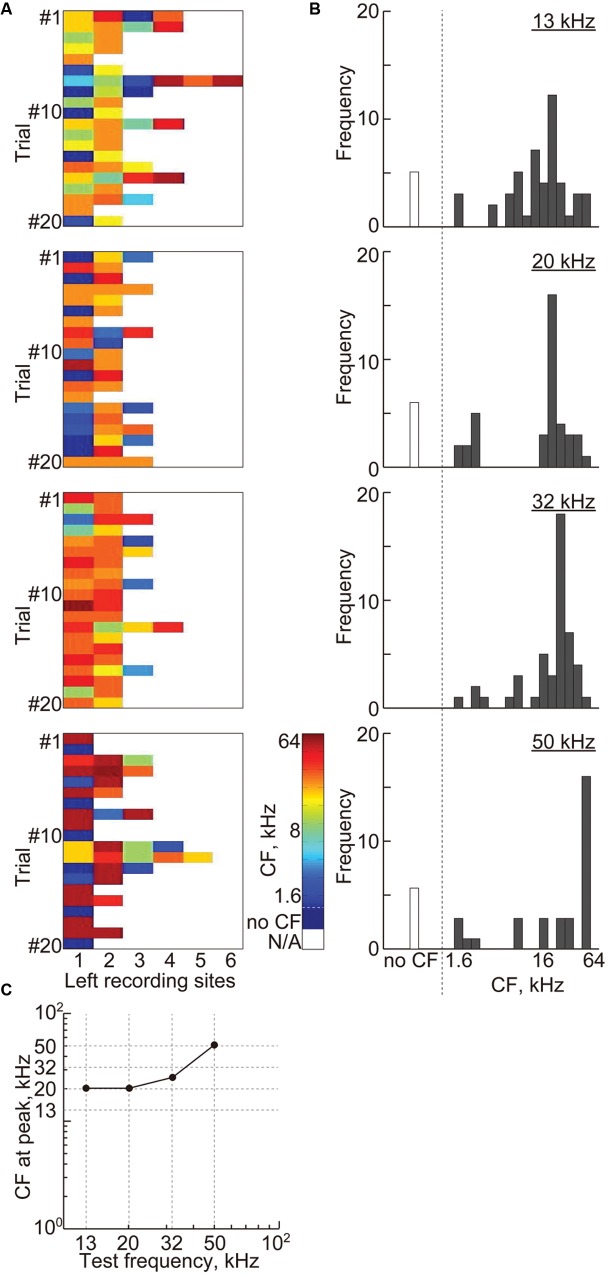
**Cortical representation of transient activities based on characteristic frequencies.** **(A)** Each color shows the CF at the remaining recording sites with non-zero weights in the most successful 20 discrimination trials of each test frequency, i.e., 13, 20, 32, and 50 kHz. **(B)** The histograms of the CF shown in **(A)** with respect to the 18 frequencies ranging from 1.6 to 64 kHz. **(C)** The CFs at the peak of the histograms with respect to the test frequencies.

For transient activity, 2.3 ± 0.96 (mean ± SD) recording sites remained, and **Figure 8A** shows the CFs at these recording sites. The histograms of the CFs show that most of these remaining sites, i.e., 90.8%, had CFs, which gradually increased with the test frequency (**Figure 8B**). The CFs at the peaks of these histograms almost corresponded to the test frequencies (**Figure 8C**), indicating the consistency of the frequency representation of the AEP and the tonotopic organization in the auditory cortex.

**FIGURE 9:**
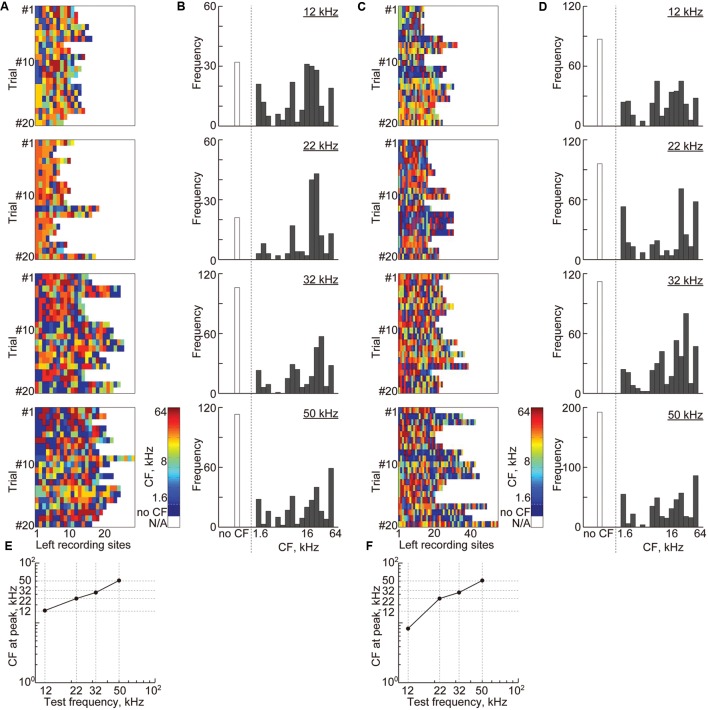
**Cortical representation of sustained activities based on characteristic frequencies.** **(A)** Each color shows the CF at the remaining recording sites with non-zero weights in the most successful 20 discrimination trials of each test frequency from the high-gamma power. **(B)** The histograms of the CF shown in **(A)** with respect to the 18 frequencies ranging from 1.6 to 64 kHz. **(C)** Each color shows the CF at recording sites of the remaining features in the most successful 20 discrimination trials of each test frequency from the high-gamma PLVs. **(D)** The histograms of the CF shown in **(C)**. **(E,F)** The CFs at the peak of the histograms with respect to the test frequencies.

The same tendency was observed in the sustained activities (**Figure 9**). For high-gamma band-power, 15 ± 5.9 recording sites remained, and for PLV, 12.5 ± 4.3 pairs of recording sites (i.e., 25 ± 8.6 recording sites) remained (**Figures 9A,C**). Compared to transient activities, these sites were more likely to belong to the non-auditory region (i.e., 22.6 and 24.3% for band-power and PLV, respectively, compared to 9.2% for AEPs). However, the remaining sound-responsive sites had CFs that were close to the decoded test frequency (**Figures 9B,D–F**), indicating that the frequency representation of the band-power and PLV also corresponds to the tonotopic organization.

**FIGURE 10:**
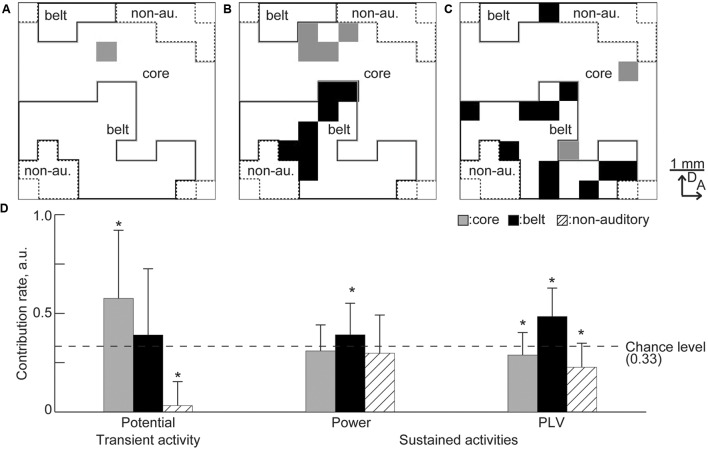
**Predominance of the cortical region in the decoding.** **(A–C)** Representative spatial distribution of the remaining recording sites in the decoding from **(A)** the transient activity, **(B)** high-gamma power, and **(C)** high-gamma PLV. Gray and black squares indicate the remaining recording sites. In these representative results, there was no remaining site in the non-auditory region. **(D)** Contribution rate for each activity. Asterisks indicate that contribution rate was better than the chance level, i.e., 0.33: ^∗^*p* < 0.05 (Mann–Whitney *U* test with Bonferroni correction for three comparisons).

The remaining sites also revealed that the frequency representation of transient and sustained activities depend on the specific auditory region. **Figures 10A–C** shows the representative maps of the remaining recording sites. More recording sites in the belt area remained for the sustained activities than for the transient activities. To quantify regional dominance, i.e., numerical advantage, the contribution rate was calculated in each region by computing the proportion of remaining sites in each area and normalizing with respect to the summation of the proportions in the core, belt, and non-auditory regions. For example, in **Figure 10B**, the proportions of the remaining sites in the core, belt, and non-auditory regions were 4/44, 7/37, and 0/15, and the contribution rates were 0.32, 0.68, and 0, respectively. Therefore, random selection of the informative recording sites will result in contribution rate at 0.33. Consequently, for transient activity, the contribution rate of the core region and the non-auditory region were, respectively, higher and lower than the chance level, i.e., 0.33 [**Figure 10D**, *n* = 20 (trials), *p* < 0.05 (Mann–Whitney *U* test with Bonferroni correction for three comparisons)], suggesting that transient activity depends on the core region. In contrast, the contribution rate for sustained activities was higher in the belt region than the chance level (**Figure 10D**), indicating belt-region dominance for sustained activity. Taken together, the remaining recording sites revealed that both transient and sustained activities represent the test frequency according to the tonotopic organization of the auditory cortex, but depend on different cortical regions.

### Decoding of Test Frequencies in Several Frequency Ranges

**FIGURE 11:**
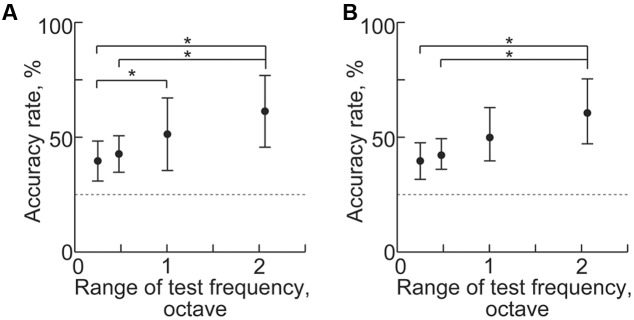
**Decoding of test frequencies in several frequency ranges.** **(A,B)** Decoding accuracy in **(A)** high-gamma power and **(B)** PLV with respect to the range of the four test frequencies (shown in **Table 1**). Asterisks indicate statistical significance: ^∗^*p* < 0.05 (Wilcoxon signed-rank test).

Machine learning from transient activities fails to discriminate neighboring test frequencies because sparse recording cannot generate distinct patterns from close test frequencies. We found that the frequency representation of sustained activities also was disorganized when the test frequencies were too close. As the range of the four test frequencies narrowed, decoding accuracies significantly decreased for both band-power and PLV [**Figure 11**, *n* = 8 (rats), *p* < 0.05 (Wilcoxon signed-rank test)]. These results suggest that the distance of the test frequencies strongly affects difference in the spatial patterns of the sustained activities. Thus, the frequency representation of sustained activities is based on the tonotopic organization of the auditory cortex.

## Discussion

In this study, we investigated whether and how sustained activities represent sound information by decoding test frequencies from densely mapped neural activities in the rat auditory cortex using SLR. SLR successfully discriminated spontaneous and sound-induced activities from band-specific power and PLV in sustained activities and AEPs in transient activities. SLR performed particularly well on high-gamma band data (**Figures 5** and **6**). In addition, informative recording sites selected by the SLR algorithm revealed that both transient and sustained activities represent sound information in a tonotopic manner (**Figures 8** and **9**). Yet, transient and sustained activities receive their main contributions from different cortical fields (**Figure 10**). Finally, discrimination of close test frequencies from sustained activities resulted in a lower decoding accuracy, indicating that the place code of sound frequency is conserved in sustained activities (**Figure 11**). Taken together, our decoding-based analysis provides compelling evidence that long-lasting sustained activities also represent sound information in a tonotopic and field-specific manner.

### Methodological Considerations

In the present study, SLR was able to decode sound information from densely recorded transient and sustained activities, including data with dimensionality (e.g., 96 or 4,560 dimensions) that sometimes exceeded the number of samples (120–240). To decode high-dimensional data with sparse representation, SLR utilizes automatic relevance determination which provides an effective method for pruning irrelevant features. Previous studies have demonstrated that SLR maintains adequate decoding performance for simulations in which the input dimensions were 20 times the number of samples, and that SLR could classify functional magnetic resonance imaging (fMRI) data when its input dimension was 20 to 100 times that of the number of samples (Miyawaki et al., 2008; Yamashita et al., 2008). In the present study, SLR exhibited sufficient performance for input dimensions that were 0.5–23 times the number of samples, which is consistent with previous studies and suggests the sparse representation of these neural activities.

Informative recording sites selected by SLR demonstrated the tonotopic and field-specific representation of transient and sustained activities. However, there are two issues to address regarding the relevant recording sites in the non-auditory region and the slight discrepancy observed between the CF and test frequency at relevant recording sites. For transient activities, SLR selected 2.3 relevant recording sites on average, 90% of which had CF. For sustained activities, SLR selected 13 relevant recording sites from band-specific power and 15 relevant recording sites from PLV; a quarter of these belonged to the non-auditory region, which may be due to methodological issues in machine learning and recording. In a previous simulation, approximately 45% of the features selected by SLR were irrelevant, which suggests that the selected features in the non-auditory region in our current study also are irrelevant (Yamashita et al., 2008). The selection of features from the non-auditory region may also be due to the large spatial decay of LFP. The spatial resolution of the microelectrode array, i.e., 400 μm, was as large as the spatial decay of LFPs, i.e., 500 μm (Gray et al., 1995; Frien and Eckhorn, 2000; Noreña and Eggermont, 2002). Therefore, recording sites in the non-auditory region may record weak AEPs that are actually elicited from the neuronal population surrounding the neighboring recording sites and SLR may select such weak AEPs as relevant features.

The spatial resolution of our recordings and the spatial decay of LFP may also explain the slight discrepancy observed between the CF and test frequency at relevant recording sites. For example, rat A1 has an approximately 3,600-μm anterior-to-posterior tonotopic axis, covering six octaves, i.e., 1–64 kHz. Therefore, the CF difference between neighboring recording sites is 0.7 octaves, which is comparable to the discrepancy between the test frequency and the CF at the relevant recording sites determined from the transient AEP (0.6 octaves, 13 kHz vs. 20 kHz) and from the PLV (0.6 octaves, 12 kHz vs. 8 kHz). Such limitations of the recording technique should be taken into account when decoding test frequencies from neural activity patterns.

It should be noted that it is difficult to interpret weights or informative recording sites derived from machine learning, especially in the reconstruction of true signal component pattern, e.g., spatial, spectral or temporal pattern, from ‘raw’ weights, because decoding processes sometimes set non-zero weight at non-informative site to cancel out noise (Haufe et al., 2014). To cope with such difficulties, we pooled the informative sites irrespective of their weights and investigated most often-selected CF and subregion, demonstrating tonotopic and field-specific representation. Previous fMRI study also pooled informative functional connection and investigated their characteristics, i.e., hemispheric distribution, to discuss possible neural correlates (Yahata et al., 2016). Taken together, there is a possibility of investigating neural representation from remaining features, although the limitation of the machine learning should be carefully considered.

### Neural Mechanisms Underlying Transient and Sustained Activities

Sparse logistic regression successfully discriminated transient AEPs from the spontaneous activities and revealed their tonotopic representation (**Figures 5** and **8**). LFPs recorded from the auditory cortex reflect excitatory and inhibitory synaptic inputs to cortical neurons around the recording sites, and the fourth layer of the auditory cortex receives excitatory inputs mainly from the auditory thalamus. Onset of an acoustic stimulus elicits synchronous activities in the cochlear hair cells and auditory nerve, which provides transient activity with a high signal-to-noise ratio and a place code of sound frequency. These synchronized onset activities are effectively and tonotopically transmitted through the ascending pathways to the auditory cortex. Therefore, cortical transient activities, or AEPs, are robust enough for machine learning to discriminate such sound-induced activities from spontaneous activities with weak and unstable fluctuations. Moreover, machine learning can easily select relevant features from clear tonotopic patterns of AEPs, as recording sites are located in the activation foci of the spatial pattern of AEPs.

This study also demonstrated that sound-induced sustained activities represent sound information in a tonotopic manner (**Figures 5**, **6**, **9**, and **11**), indicating that the auditory cortex continues to receive ascending neural activities from the auditory periphery even after neural activities seem to adapt to long-lasting sounds. After tens of milliseconds from the sound onset, the auditory nerves sustain steady-state firing activity, although these firing rates are reduced by approximately half (Smith and Zwislocki, 1975; Smith, 1979; Smith and Brachman, 1980; Westerman and Smith, 1984). Such steady-state firings are also tonotopically forwarded to the auditory cortex, and this constant synaptic input may affect band-specific power in cortical LFPs. Moreover, PLV also may reflect steady-state ascending inputs. In the auditory thalamus, some neurons tonotopically project to both A1 and AAF (Lee et al., 2004; Lee and Winer, 2005). Although neurons with branched axons to these subfields are scarce and it has not been demonstrated whether there are any thalamic neurons projecting to both the core and belt regions, such common ascending inputs from the thalamus could mediate distinct phase synchrony between distant neural populations, especially in sustained activities after adaptation as well as the firing synchrony investigated in previous studies (DeCharms and Merzenich, 1996; Eggermont, 1997).

For both band-specific power and PLV, the highest decoding performance was achieved in the high-gamma band (**Figures 5** and **6**), which mainly reflects activities of the cortical inhibitory interneurons (Jefferys et al., 1996; Hasenstaub et al., 2005; Bartos et al., 2007). Previous studies indicated that cortical inhibition plays an important role in both sparse coding (Hromádka et al., 2008; Wu et al., 2008; Wolfe et al., 2010) and synchronous activities in the cortical network (Jefferys et al., 1996; Singer, 1996; White et al., 1998; Hasenstaub et al., 2005). Moreover, there is some evidence that cortical oscillatory activity in the gamma band reflects auditory perception (Crone et al., 2001; Weisz et al., 2007), and mediates feature binding in visual perception (Kreiter and Singer, 1996). Therefore, our results indicate that cortical inhibitory interneurons contribute to the sparse tonotopic representation of sound frequency in sustained cortical activity and to the synchronous activities that may mediate auditory perception.

Finally, our results demonstrated that the sound representation of transient and sustained activities depend on the core and belt regions, respectively (**Figure 10**), indicating that higher-order auditory areas predominate over lower-order areas in sustained activities and vice versa in transient activities. For transient activities, strong feedforward processing from the ventral division of the auditory thalamus to the core region dominates the clear cortical representation of sound frequency and intensity (Takahashi et al., 2005; Lee and Winer, 2008), which was also decodable in the previous study (Funamizu et al., 2011). However, supplemental information in sound can be represented in sustained activities in the belt region through the feedforward projection from the dorsal and medial division of the auditory thalamus (Roger and Arnault, 1989; Arnault and Roger, 1990; Malmierca, 2003; Lee and Winer, 2008), which are involved in higher order function, e.g., attention and emotional learning. In addition, it is possible that sustained activities in the belt region reflects its reciprocal projections that spread widely throughout the brain, including in the basal ganglia, amygdala, and prefrontal cortex (Reale and Imig, 1983; Roger and Arnault, 1989; Arnault and Roger, 1990; LeDoux et al., 1991; Mascagni et al., 1993; Romanski and LeDoux, 1993; Romanski et al., 1999a,b; Sah et al., 2003; Winer, 2006). Recent studies also support that the secondary auditory cortex contributes to sound-associated memory retrieval (Sacco and Sacchetti, 2010) and that sound-associated emotion affects sustained activities (Shiramatsu et al., 2014). Taken together, our results indicate that the auditory cortex processes sound information tonotopically and in an hierarchical manner; transient activities mainly represent basic sound information, i.e., frequency and intensity, whereas sustained activities represent supplemental information, including sound-associated emotion and memory in addition to sound frequency.

## Author Contributions

TS and HT designed the research and interpreted the results. TS, TN, and HT performed the experiments. TS and KA analyzed the data. TS prepared the figures, and drafted the manuscript. HT revised the manuscript. TS, TN, KA, and HT approved the final version of the manuscript.

## Conflict of Interest Statement

The authors declare that the research was conducted in the absence of any commercial or financial relationships that could be construed as a potential conflict of interest.

The reviewer HT and handling Editor declared their shared affiliation, and the handling Editor states that the process nevertheless met the standards of a fair and objective review.

## Abbreviations

A1: primary auditory cortex
AAF: anterior auditory field
AEP: auditory evoked potential
AVAF: anterior ventral auditory field
CF: characteristic frequency
DAF: dorsal auditory field
FRA: frequency responsive area
LFP: local field potential
MUA: multi-unit activities
PAF: posterior auditory field
PLV: phase locking value
PSTH: post-stimulus time histograms
SLR: sparse logistic regression
SRAF: suprarhinal auditory field
VAF: ventral auditory field
