# Supplementary material for: Tonotopic and Field-Specific Representation of Long-Lasting Sustained Activity in Rat Auditory Cortex
Source: Front Neural Circuits. 2016 Aug 10;10:59. doi: 10.3389/fncir.2016.00059 (PMC4978722; doi:10.3389/fncir.2016.00059)
Supplement: Supplementary file 1 [file Image_1.PDF]

Supplementary Material

Tonotopic and Field-Specific Representation of Long-Lasting Sustained Activity in Rat Auditory Cortex

Tomoyo I. Shiramatsu, Takahiro Noda, Kan Akutsu and Hirokazu Takahashi*

*** Correspondence:** Hirokazu Takahashi: takahashi@i.u-tokyo.ac.jp

1 Supplementary Figures

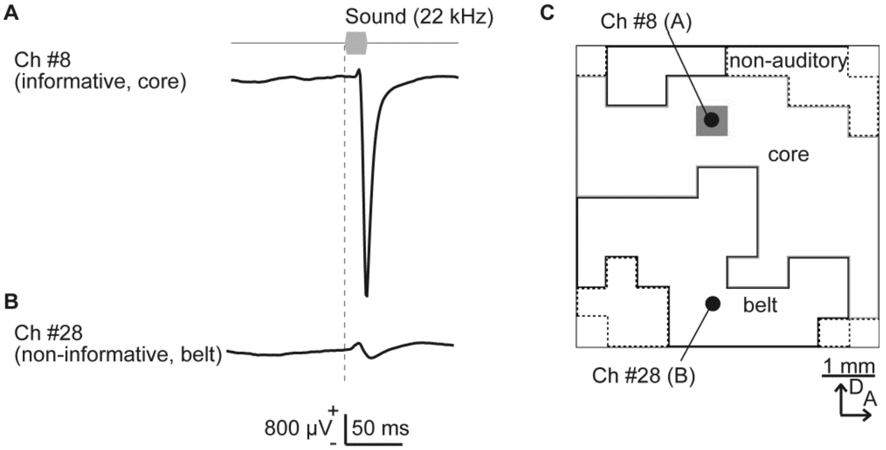

Supplementary Figure 1. Representative transient activities in the informative and non-informative recording sites. (A–B) Representative raw traces of transient local field potential (LFP) in (A) informative recording site in the core region and (B) non-informative recording site in the belt region. These responses were obtained from 22 kHz tones. (C) Arrangement of the recording sites indicated in (A, B). This figure is same as Figure 10A. One of these sites (Ch #8) was selected as an informative site by SLR during supervised learning, and the other site (Ch #28) was eliminated as a non-informative site.

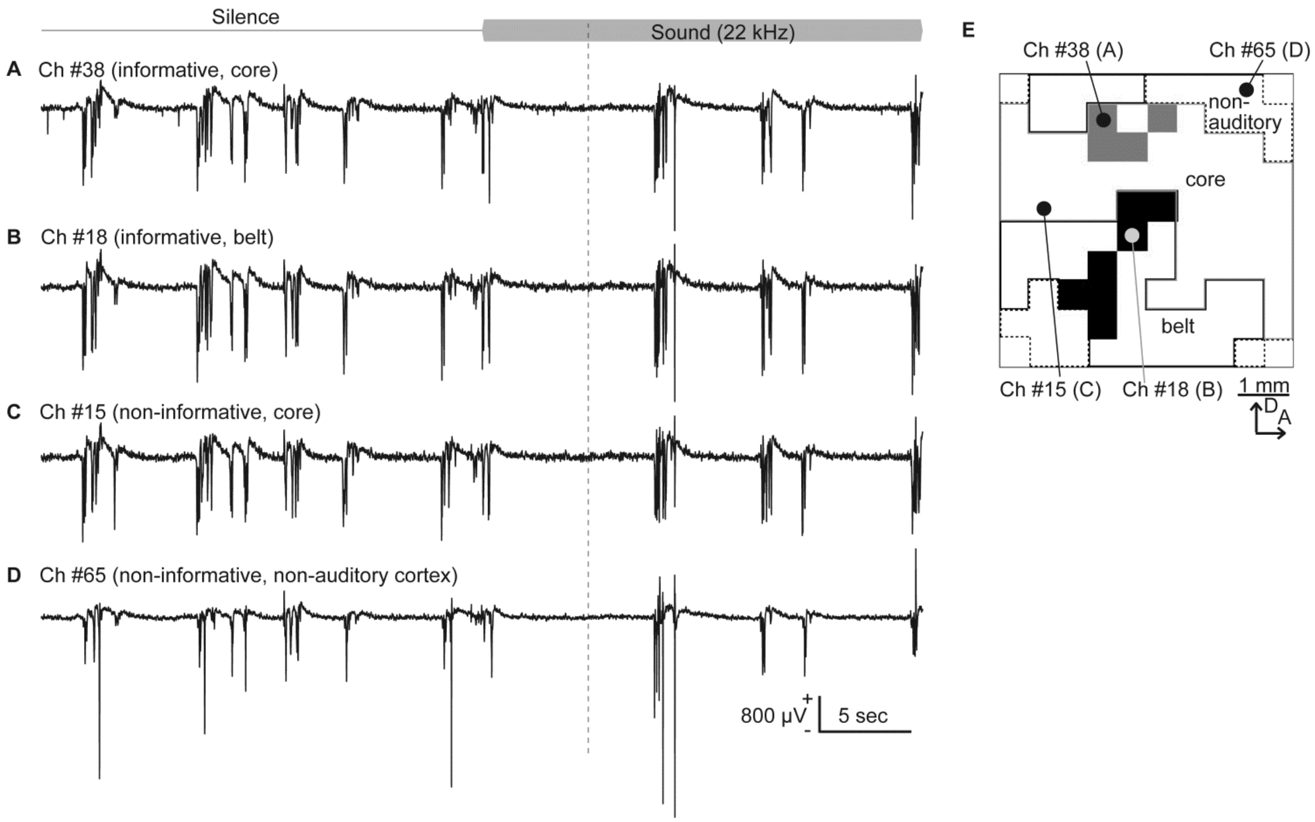

Supplementary Figure 2. Representative sustained activities in the informative and non-informative recording sites. (A–D) Representative raw traces of long-lasting, sustained local field potential (LFP) without and with sound presentation. (E) Arrangement of the recording sites indicated in (A–D). This figure is same as Figure 10B. Two of these sites (A, B) were selected as informative sites by SLR during supervised learning, and the other two sites (C, D) were eliminated as non-informative sites.

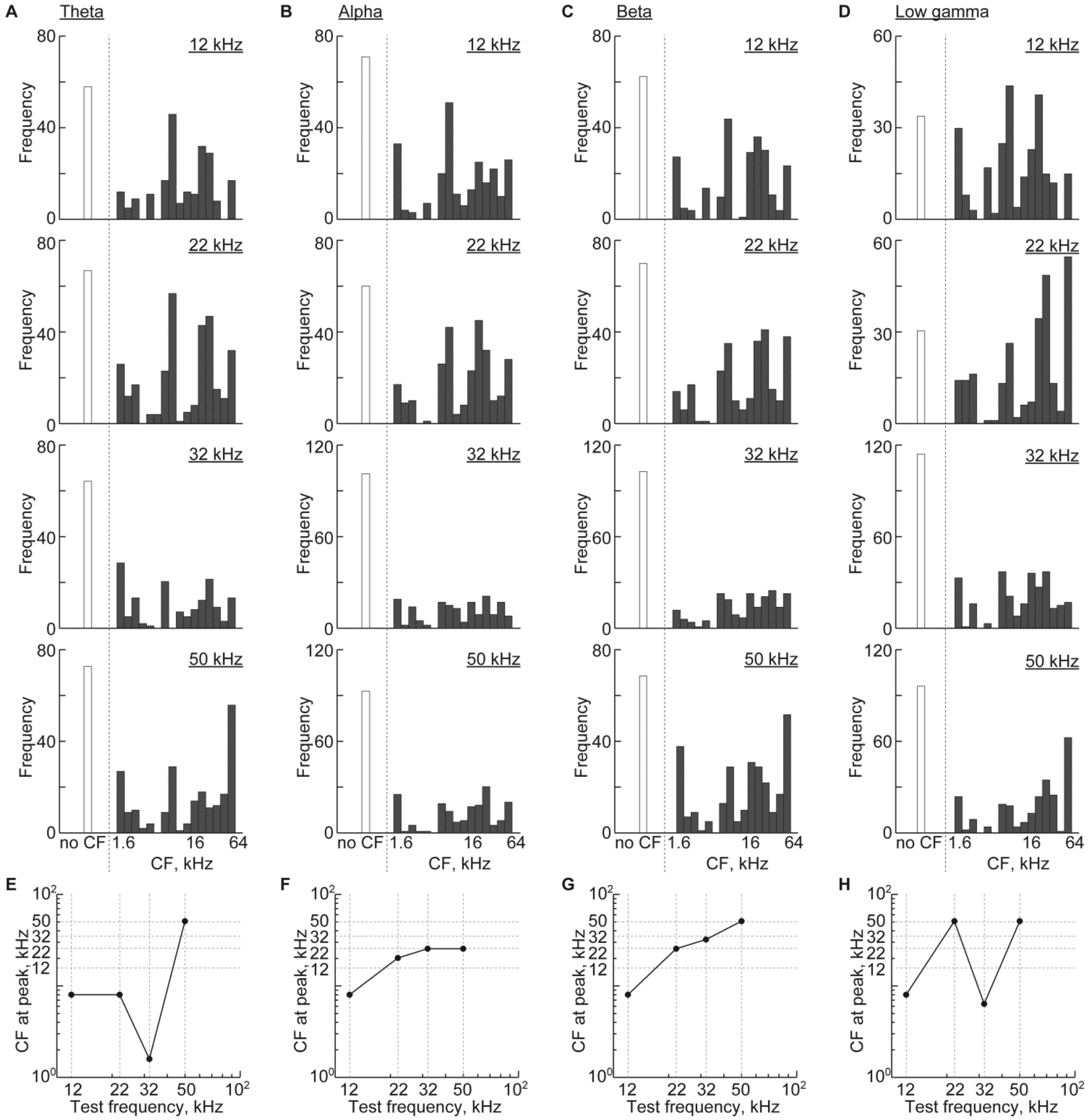

Supplementary Figure 3. Cortical representation of band specific power based on characteristic frequencies. (A–D) The histograms of the CF at the remaining recording sites with non-zero weights in the most successful 20 discrimination trials of each test frequency from (A) theta, (B) alpha, (C) beta and (D) low-gamma power. (E–H) The CFs at the peak of the histograms with respect to the test frequencies.

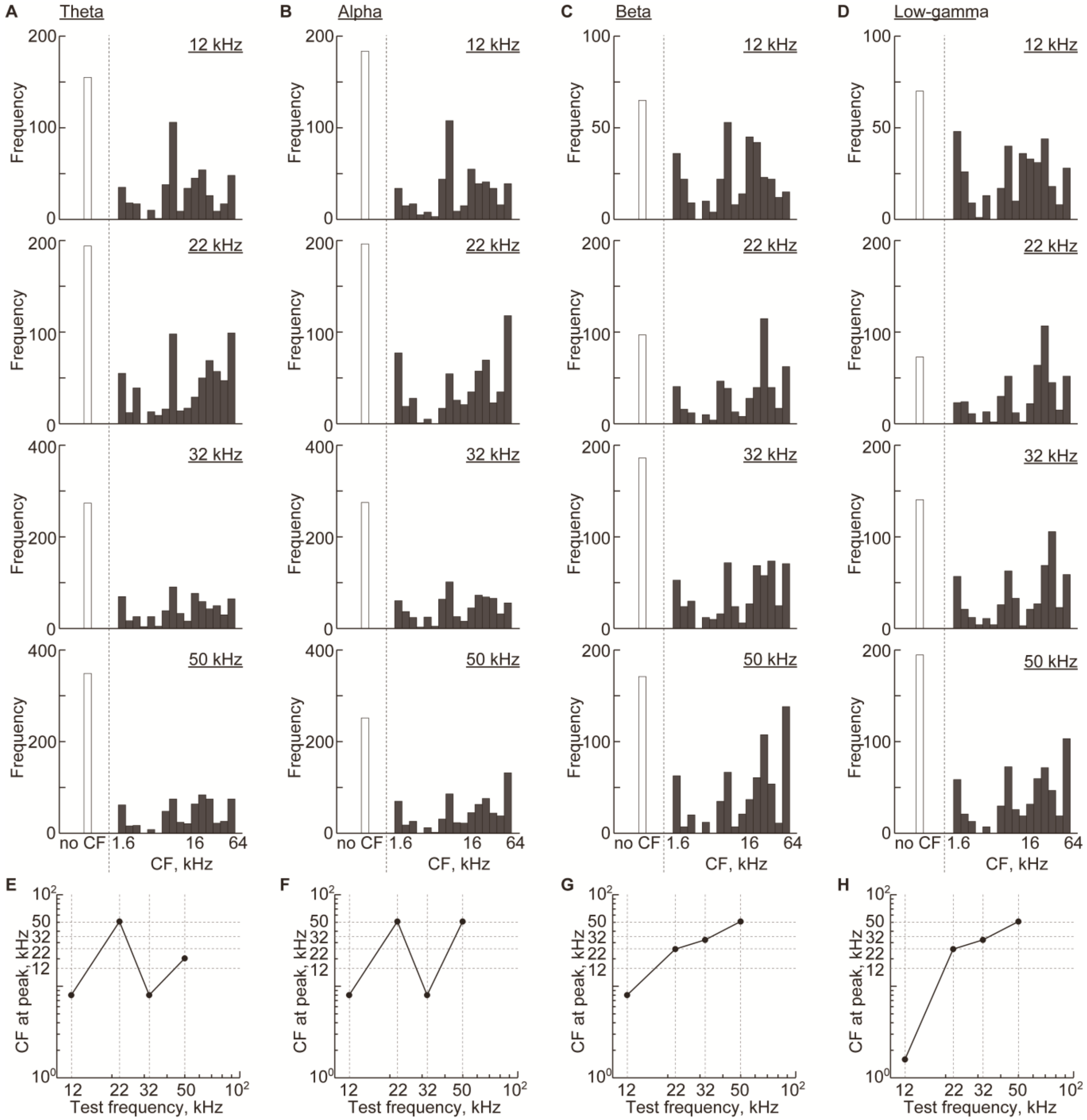

Supplementary Figure 4. Cortical representation of PLV based on characteristic frequencies. (A–D) The histograms of the CF at the remaining recording sites with non-zero weights in the most successful 20 discrimination trials of each test frequency from (A) theta, (B) alpha, (C) beta and (D) low-gamma PLV. (E–H) The CFs at the peak of the histograms with respect to the test frequencies.

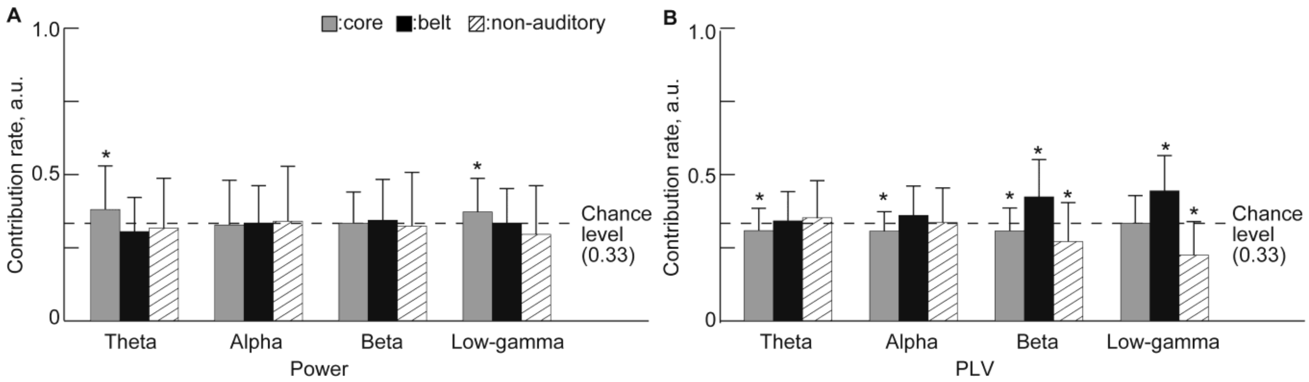

Supplementary Figure 5. Predominance of the cortical region in the decoding. (A–B) Contribution rate for (A) band-specific power and (B) PLV at each frequency band. Asterisks indicate that contribution rate was better than the chance level, i.e., 0.33: * $p < 0.05$ (Mann-Whitney *U* test with Bonferroni correction for three comparisons).
